# No Gold Standard Estimation of the Sensitivity and Specificity of Two Molecular Diagnostic Protocols for *Trypanosoma brucei spp*. in Western Kenya

**DOI:** 10.1371/journal.pone.0008628

**Published:** 2010-01-07

**Authors:** Barend Mark de Clare Bronsvoort, Beatrix von Wissmann, Eric Maurice Fèvre, Ian Graham Handel, Kim Picozzi, Sue Christina Welburn

**Affiliations:** 1 Centre for Infectious Diseases, Royal (Dick) School of Veterinary Studies, University of Edinburgh, Edinburgh, United Kingdom; 2 Centre for Infectious Diseases and Centre for Infection, Immunity and Evolution, School of Biological Sciences, University of Edinburgh, Edinburgh, United Kingdom; 3 The Roslin Institute and The Royal (Dick) School of Veterinary Studies, University of Edinburgh, Roslin, Midlothian, United Kingdom; New York University School of Medicine, United States of America

## Abstract

African animal trypanosomiasis is caused by a range of tsetse transmitted protozoan parasites including*Trypanosoma vivax, Trypanosoma congolense* and *Trypansoma brucei*. In Western Kenya and other parts of East Africa two subspecies of *T. brucei, T.b. brucei* and the zoonotic*T.b. rhodesiense*, co-circulate in livestock. A range of polymerase chain reactions (PCR) have been developed as important molecular diagnostic tools for epidemiological investigations of *T. brucei* s.l. in the animal reservoir and of its zoonotic potential. Quantification of the relative performance of different diagnostic PCRs is essential to ensure comparability of studies. This paper describes an evaluation of two diagnostic test systems for *T. brucei* using a *T. brucei* s.l. specific PCR [1] and a single nested PCR targeting the Internal Transcribed Spacer (ITS) regions of trypanosome ribosomal DNA [2]. A Bayesian formulation of the Hui-Walter latent class model was employed to estimate their test performance in the absence of a gold standard test for detecting *T.brucei* s.l. infections in ear-vein blood samples from cattle, pig, sheep and goat populations in Western Kenya, stored on Whatman FTA cards. The results indicate that the system employing the *T. brucei* s.l. specific PCR (Se_1_ = 0.760) had a higher sensitivity than the ITS-PCR (Se_2_ = 0.640); both have high specificity (Sp_1_ = 0.998; Sp_2_ = 0.997). The true prevalences for livestock populations were estimated (p_cattle_ = 0.091, p_pigs_ = 0.066, p_goats_ = 0.005_,_ p_sheep_ = 0.006), taking into account the uncertainties in the specificity and sensitivity of the two test systems. Implications of test performance include the required survey sample size; due to its higher sensitivity and specificity, the *T. brucei* s.l. specific PCR requires a consistently smaller sample size than the ITS-PCR for the detection of *T. brucei* s.l. However the ITS-PCR is able to simultaneously screen samples for other pathogenic trypanosomes and may thus be, overall, a better choice of test in multi-organism studies.

## Introduction

Trypanosomiasis, or ‘nagana’, is an infectious disease of livestock caused by a range of protozoan parasites. *Trypanosoma vivax, Trypanosoma congolense* and *Trypansoma brucei* are the three most important species of trypanosome, responsible for considerable production losses and livestock morbidity where they occur [3]. These parasites are transmitted by tsetse flies in the genus *Glossina*, in which they have obligate life cycle stages. Severity of infection with these trypanosomes depends on a range of factors; in local zebu cattle (*Bos indicus*) in western Kenya and elsewhere in East Africa, trypanosomiasis is an endemic disease, causing chronic anaemia [4], enlarged lymph nodes, staring coat, weakness and depression, and general loss of productivity and overall condition, including reduced milk yield and impaired fertility [5]. *T. brucei*, which is perhaps the least pathogenic of the three species in cattle [6], has three sub-species, namely *T.b. brucei, T.b. gambiense* and *T.b. rhodesiense*; in Western Kenya and other parts of East Africa, *T.b. brucei* and *T.b. rhodesiense* co-circulate in cattle and other livestock species. As *T.b. rhodesiense* is the agent of the zoonotic form of human sleeping sickness, understanding the epidemiology of *T. brucei* s.l. in cattle is important both for understanding and controlling animal trypanosomiasis, but also with regards to estimating the size of the reservoir of human infective parasites.

Classical estimates of sensitivity and specificity are based on direct, empirical comparisons of test outcomes for different tests, where an index test is compared to an established “gold-standard” which has an assumed sensitivity and specificity of 100%. For field diagnosis in rural settings, microscopy-based techniques using direct observation of wet blood films, or concentration techniques such as the Buffy Coat Technique, BCT [7] and the Haematocrit Centrifugation Technique, HCT [8] are the most common method of parasite detection, and have historically been considered the gold standard. Recent studies [9] have illustrated, however, that microscopy has a very poor sensitivity compared to diagnosis with molecular tools, highlighting that previous studies using these technologies are likely to have significantly underestimated both animal- and herd-level prevalence of these pathogens. This has clinical implications for the management of individual animals, but also important epidemiological implications regarding the zoonotic potential of *T. brucei* s.l. As a result, PCR-based diagnosis of *T. brucei* s.l. in livestock has now been used in a number of studies across Africa [2], [10], [11], [12], [13], using a number of different protocols and methods [1], [2], [14].

To enable comparisons between different studies, the relative performance of different testing systems needs to be quantified, preferably in such a way as to enable unbiased estimates of the true prevalence to be made, while accounting for uncertainty in the specificity and sensitivity of the system used (we refer to the *testing system* as the combination of the diagnostic protocol *and* method of sample collection and processing). We know of only one other study [15] that compared different PCR protocols (including those we examine here); while this was a valuable addition to the literature, their analysis assessed only agreement between tests and did not assess sensitivity or specificity, or indeed make estimates of the true prevalence based on the outcomes of the different tests. The development of a latent class model by Hui and Walter [16] to estimate sensitivity and specificity avoids the need for a “gold standard” which is rarely, if ever, genuinely perfect [17]. The extension of this into a Bayesian framework allows the uncertainty in the prior beliefs about the tests to be included [18] and full posterior distributions of the estimates to be given.

In the present paper, we compare two PCR-based testing systems for the detection of *T. brucei* s.l. in populations of cattle, pigs, sheep and goats in Western Kenya: a) a *T. brucei* s.l. specific primer pair [1] on material originating from ear-vein blood and stored on Whatman FTA filter cards [9]; b) ear-vein blood samples on Whatman FTA cards amplified using a single nested PCR targeting the Internal Transcribed Spacer (ITS) regions of ribosomal DNA [2]. We present estimates of sensitivity, specificity and predictive values for these two testing systems following the STARD guidelines [19] and report on the estimated true prevalence of *T. brucei* s.l. in livestock in two areas within the Busia District of Western Kenya.

## Results

A total of 1,260 cattle, 764 goats, 311 pigs and 427 sheep were sampled across the two study sites and tested using both PCR techniques. The estimated apparent prevalence of *T. brucei* s.l. by each PCR method as well as the cumulative apparent prevalence are given in Table 1. The apparent prevalence is highest in cattle and pigs and lowest in sheep and goats. The estimates from each test differ only slightly, with the ITS-PCR appearing to be less sensitive than the *T. brucei* s.l. specific PCR. In this situation of low prevalence this difference in apparent prevalences is unlikely to biologically important. However, the different tests are clearly classifying slightly different subsets of the population as infected/uninfected, highlighted by the cumulative prevalence being higher than the individual estimates (Table 1). The species concordant and discordant test classification results are given in Table 2: these form the input for the Hui-Walter model.

**Table 1:** The apparent prevalence estimates for each species in the study based on the individual and cumulative test results from the *T. brucei* s.l. specific PCR, *T. brucei* s.l. ITS-PCR.

	T_1_+ prevalence	(95%CI)	T_2_+ prevalence	(95%CI)	Cummulative T_1_ & T_2_	(95%CI)
Cattle (n = 1260)	0.071	(0.058–0.086)	0.060	(0.048–0.075)	0.087	(0.072–0.104)
Goats (n = 764)	0.005	(0.002–0.013)	0.004	(0.001–0.011)	0.008	(0.004–0.017)
Pigs (n = 311)	0.051	(0.032–0.082)	0.045	(0.027–0.074)	0.061	(0.039–0.093)
Sheep (n = 427)	0.002	(0.0001–0.013)	0.007	(0.002–0.020)	0.007	(0.002–0.020)

T_1_ = *T. brucei* s.l. specific PCR; T_2_ = *T. brucei* s.l. ITS-PCR.

**Table 2:** Test cross tabulation by species (T_1_ = *T. brucei* s.l. specific PCR; T_2_ = *T. brucei* s.l. ITS-PCR).

	T_1_+/T_2_+	T_1_−/T_2_+	T_1_+/T_2_−	T_1_−/T_2_−
Cattle (n = 1260)	55	21	34	1150
Goats (n = 764)	1	2	3	758
Pigs (n = 311)	11	3	5	292
Sheep (n = 427)	1	2	0	424

**Figure 1:**
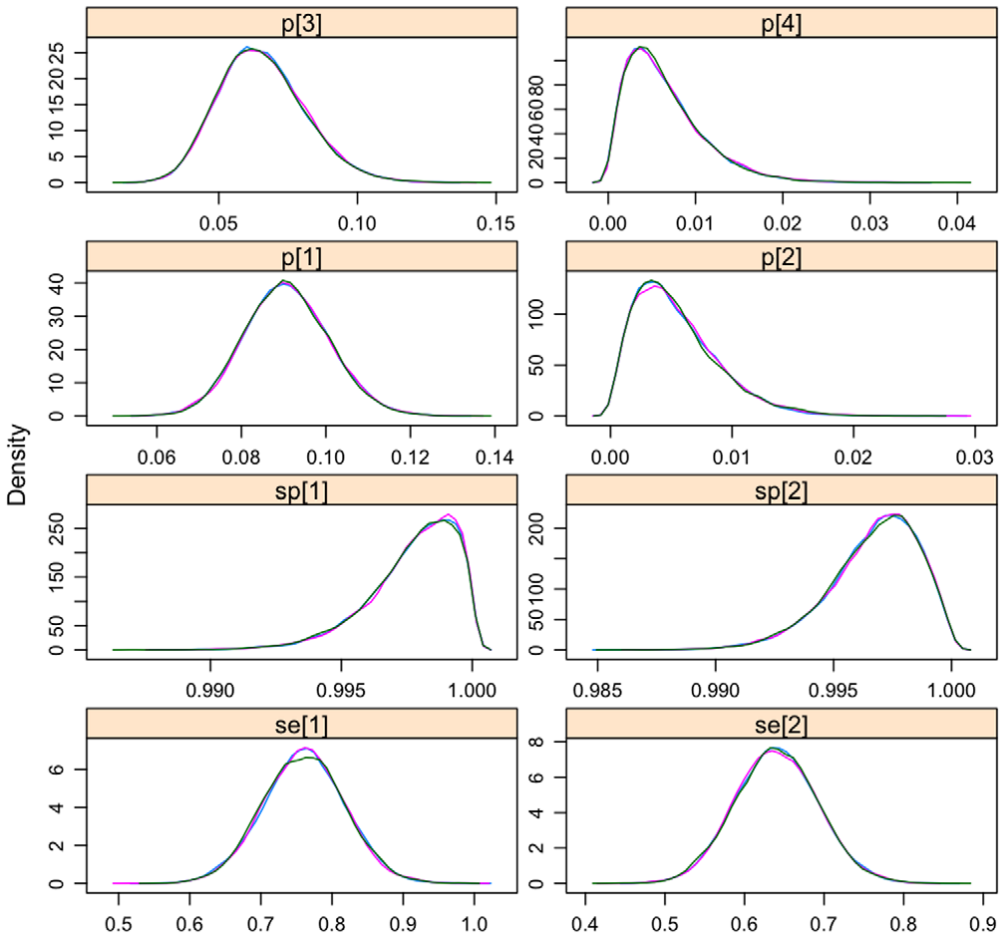
Probability density plots. Probability density plots for each test parameter estimate for the *T. brucei* s.l. specific PCR (sensitivity = Se[1]; specificity = Sp[1]), *T. brucei* s.l. ITS-PCR (sensitivity = Se[2]; specificity = Sp[2]) and the adjusted prevalence estimates from the Hui-Walter model assuming conditional independence for cattle (p[1]), goats (p[2]), pigs (p[3]) and sheep (p[4]). The x axes give the parameter estimate and the y axis the relative likelihood of it taking that value.

**Figure 2:**
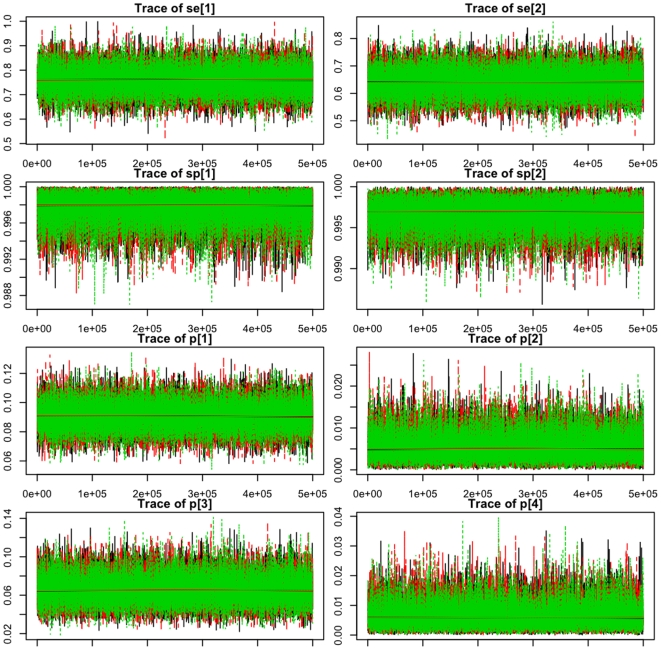
Trace plots. MCMC history plots for each parameter of the Hui-Walter model for parameter estimates and true prevalence estimates for the *T. brucei* s.l. specific PCR (sensitivity = Se[1]; specificity = Sp[1]), *T. brucei* s.l. ITS-PCR (sensitivity = Se[2]; specificity = Sp[2]), cattle (p[1]), goats (p[2]), pigs (p[3]) and sheep (p[4]). The plots record every 10th sample from 500,000 iterations and the x axis is the sequence of iterations and the y axis the parameter value from that iteration.

The unbiased estimates from the Hui-Walter model are given in Table 3. The estimates of sensitivity are low for both tests but the *T. brucei* s.l. specific PCR on average appears to be more sensitive. Both tests are highly specific. The estimated probability distributions of the test parameters are given in the the density plots in Figure 1. These plot show the relative probability of the parameter taking a given value on the x axis and are effectively a smoothed histogram. The parameter value at the peak of the distribution represents the most likely value. The model behaved well with good mixing of the three chains as seen in the trace plots (Figure 2) which shows the samples for three chains for each parameter. They show that the chains are statistically stationary and are not autocorrelated. The Gelman-Rubin potential scale reductiom factor (PSRF) statistic for all parameters was <1.05. The PSRF is a measure of MCMC chain convergence and values substantially above 1 indicate lack of convergence [20]. Both tests have very high specificity with only moderate sensitivity, although the *T. brucei* s.l. specific PCR appears to be about 12% more sensitive than the ITS-PCR. The unbiased estimates of the true prevalence in each host species (Table 3) are higher for cattle and pigs than the apparent prevalence estimates for both those species, indicating that both PCR tests normally underestimate apparent prevalence. The prevalences are so low for sheep and goats that it is difficult to draw clear conclusions for these species. The estimates for sensitivity, specificity and prevelence were robust to removal of the cattle sub-population.

**Table 3:** Parameter estimates (and Bayesian 95% credibility intervals, BCI) for the *T. brucei* s.l. specific PCR (Se_1_ and Sp_1_), *T. brucei* s.l. ITS-PCR (Se_2_ and Sp_2_) and adjusted *T. brucei* s.l. prevalence estimates for cattle (p_cattle_), goats (p_goats_), pigs (p_pigs_) and sheep (p_sheep_) from the Hui-Walter model assuming conditional independence.

Parameter	Mean	95% BCI
Se_1_	0.760	0.648–0.873
Sp_1_	0.998	0.994–1.00
Se_2_	0.640	0.540–0.744
Sp_2_	0.997	0.992–1.00
p_cattle_	0.091	0.072–0.111
p_goats_	0.005	0.001–0.014
p_pigs_	0.066	0.038–0.099
p_sheep_	0.006	0.001–0.018

**Figure 3:**
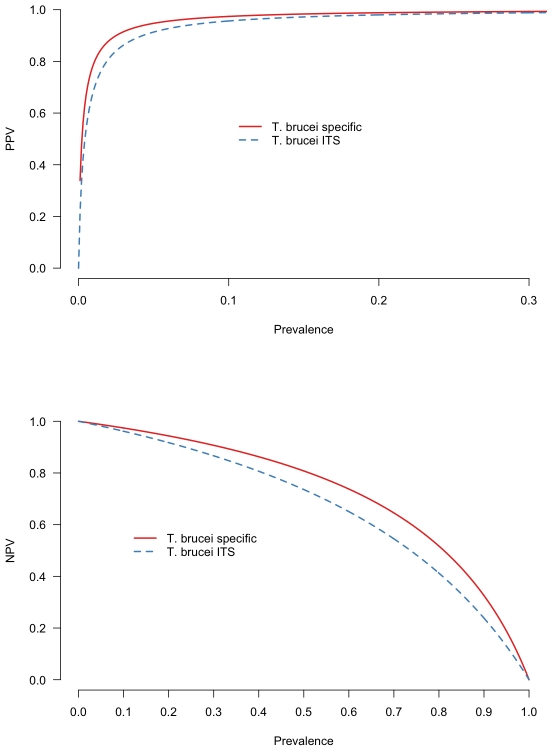
Predictive value plots. The positive (PPV) and negative predictive value (NPV) of the *T. brucei* s.l. specific PCR and the ITS PCR over a range of prevalence of *T. brucei* s.l. PPV is the positive predictive value of the test at a given prevalence i.e. how likely the animal is to have the pathogen given that it has a positive test result. NPV is the negative predictive value of the test at a given prevalence i.e. how likely is an animal not to have the pathogen given that it had a negative test result.

Having estimated the sensitivity and specificity for each test, we use these outputs to estimate the positive predictive values (PPV) and negative predictive values (NPV) for each test across a range of true prevalences (Figure 3). The PPV is the probability that an animal is truly positive given that it has had a positive test result. The NPV is exactly the inverse; that is, the probability that an animal is truly negative given that it is test negative. The distribution of PPVs and NPVs across a range of prevalences clearly shows that the test performance in this regard is related to prevalence - both tests have high PPVs above 20% prevalence, but this decreases rapidly as the prevalence decreases. Inversely the NPV are extremely high at lower prevalences but decrease steadily as the prevalence increases. For both the PPV and the NPV, the *T. brucei* s.l. specific PCR has a higher predictive value at any given prevalence than the *T. brucei* s.l. ITS-PCR. The estimated PPV and NPV for each test in each sub population are shown in Table 4. From this it is clear that in these sub populations, the PPV of both tests is very high for cattle and pigs, in the region of 95%, because of their relatively high prevalence but decreases markedly for sheep and goats to around 50% due to the low prevalence in these species. For the prevalences estimated in these sub-popuatlions, the NPVs were extremely high.

**Table 4:** Estimated predictive values of the two tests in the four sub-populations (Test 1 = *T. brucei* s.l. specific PCR; test 2 = *T. brucei* s.l. ITS-PCR).

Sub-population	Prevalence	PPV	PPV	NPV	NPV
		Test 1	Test 2	Test 1	Test 2
cattle	0.091	0.970	0.950	0.976	0.965
goats	0.005	0.625	0.505	0.999	0.998
pigs	0.066	0.957	0.929	0.983	0.975
sheep	0.006	0.659	0.534	0.998	0.998

PPV is the positive predictive value of the test at a given prevalence i.e. how likely the animal is to have the pathogen given that it has a positive test result. NPV is the negative predictive value of the test at a given prevalence i.e. how likely is an animal not to have the pathogen given that it had a negative test result.

**Figure 4:**
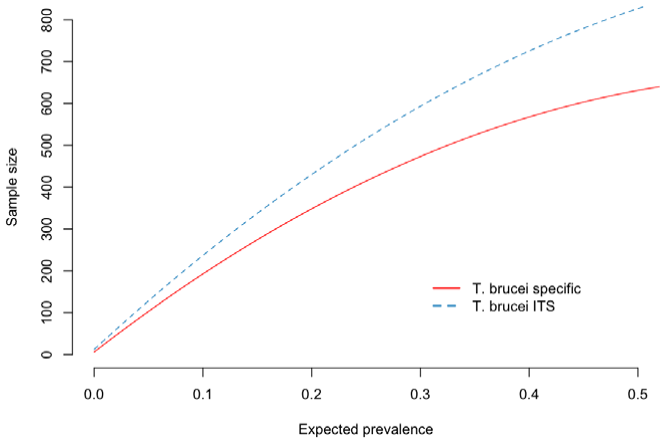
Sample size plots. Approximate sample sizes for a simple random survey to estimate infection prevalence using the two tests. The samples sizes are calcualted to estimate prevalence with a 95% confidence interval for an absolute precision of +/− 5%.

The impact of the test's performance on survey sample size are illustrated in Figure 4. The *T. brucei* s.l. specific PCR requires a consistently smaller sample size than the *T. brucei* s.l. ITS-PCR as expected with its higher sensitivity and specificity.

## Discussion

Molecular diagnostic tools, and in particular PCR, have vastly improved the detection of trypanosome infections over standard parasitological techniques, by lowering the parasitaemia detection limit by several orders of magnitude. Even when applying concentration techniques, such as the haematocrit centrifugation technique (HCT) or the buffy coat technique (BCT), the analytical sensitivity of microscopy ranges between detectable parasitaemias of 2.5×10^2^ to 5×10^3^ parasites/ml of blood depending on trypanosome species [21], whereas PCR can detect the presence of parasite DNA equivalent to one trypanosome in 10ml of host blood [22]. The analytic detection limit of the *T. brucei* s.l. specific PCR has been shown to be as low as 1/10 of the genetic material of a single trypanosome per PCR reaction [1]. The ITS - PCR was been shown to detect trypanosome DNA at a dilution equivalent to less than one parasite/ml of host blood [2]. Furthermore, primer design targeting precise DNA sequences ensures high specificity of PCR, making it independent of morphological differences required for speciation by microscopy. PCR has thus been incorporated as the diagnostic tool of choice into a wide number of studies investigating the epidemiology of trypanosomiasis, especially since advances in preservation methodology for biological samples have facilitated collection and stabilization of field samples of sufficiently high quality for molecular analysis. The field applications of PCR include estimating trypanosome prevalence for the monitoring of control programmes, though due to the cost and level of laboratory equipment involved, PCR is currently not suitable for diagnostic testing of individual animals for treatment decisions at the local level.

To enable comparison between different protocols, this study used a non-gold standard approach to quantify the parameters of the two PCR-based test systems: a *T. brucei* s.l. specific PCR [1] and ITS-PCR [2] run in parallel on different punches of the same wholeblood samples from cattle, pigs, goats and sheep stored on FTA-cards. This approach simultaneously allowed for an unbiased estimate of the true prevalence of *T. brucei* s.l. infections in the different livestock species, whilst accounting for the uncertainties in the specificity and sensitivity of the testing systems used. As expected, based on the high target-specificity of the primers designed for the respective PCRs [1], [2] the specificity of the test system was high (*T.brucei* s.l. specific PCR: Sp_1_ = 0.998; *T. brucei* s.l. ITS-PCR: Sp_2_ = 0.997) for both test systems. However, the sensitivities of the test systems were lower than expected (Se_1_ = 0.76, Se_2_ = 0.64) considering the detection limit of the PCRs themselves, which are capable of detecting very low parasitaemia. This low sensitivity may largely be due to the sample storage element of testing system - FTA cards preserve the sample by lysing cells and fixing the DNA *in situ* to the filter-paper matrix, and it has been shown that at low parasitaemias the parasite DNA is localized on the FTA card, with the result that using single punches for each PCR may result in negative PCR test results because the sub-sample (punch) of blood isolated for the specific reaction did not include any parasite DNA (Cox, PhD thesis 2007, University of Edinburgh). Other factors that may contribute to the low sensitivity of the testing system could include residual PCR inhibitors in the sampling material, such as haem, although the sample preparation protocol is designed to remove such known inhibitors [23]. Differences in sensitivity between the two testing systems may be attributable to a higher number of copies of the target sequence for the *T. brucei* s.l. specific PCR (10,000 copies/genome) as compared to the ITS-PCR (200 copies/genome) [1], [2]. It may also be that the efficiency of PCR amplifications from the FTA filter paper matrix depend on the target sequence length (1250 base pairs for the ITS-PCR and 173 for *the T. brucei* s.l. specific PCR), though there is no empirical evidence that supports such a negative effect.

The Hui-Walter model assumes that the sensitivity and specificity of the tests are the same across different populations. In this case we have used the different species as the different populations. For an antibody based test this would be problematic as the types of antibody response may be quite different between species. Here, however, the test is detecting the parasite DNA on an FTA card. While differences in parasitaemia between host species have been reported, differentiating a real species-specific effect from the expected individual animal level variation would be a significant undertaking [24], and we feel that it is safe for the purposes of this analysis to assume average parasite densities between host species are sufficiently similar to not affect this. Concern that co-infection of *T. theileri* in cattle may reduce specificity in this species was explored by re-estimation without the cattle population and estimates were found to be robust. Cross tabulation of *the T. brucei* tests with the *T. theileri* showed 27/1260 cattle and 1/427 sheep to be *T. theileri* positive. Only one cow was postive for both *T. brucei* and *T. theileri* with the ITS test. From these findings we find little evidence of *T. brucei* co-infection of *T. theileri* and therefore we conclude that specificity across the different sub-popualtions is unlikely to be influenced by *T. theileri* co-infection. The model also assumes conditional independence between the tests: i.e. given a truly positive animal, the results of the first test are independent of the second test. We believe this is justified with regards to sensitivity and specificity as the PCRs use different primer sets and target regions. The assumption that the specificity is >0.5, as reported in the methods to control the label switching issue, is justifiable as these techniques are widely accepted as highly specific because of the very nature of the technique.

The estimated true prevalences for both cattle (p_cattle_ = 0.091) and pigs (p_pigs_ = 0.066) were higher than those estimated from the results of the individual test systems, or indeed the cumulative prevalence derived for both tests (see Table 1), taking into account the uncertainties in the specificity and sensitivity. The under-estimation at the higher prevalences is a result of the low sensitivity and false negative results. The estimates in sheep (p_sheep_ = 0.006) and goats (p_goats_ = 0.005) are slightly lower than those from the cumulative test results reflecting the fact the specificity is not 1 which at very low prevalences results in low PPV for the tests and high risk of false positive results. The implications of these estimates are firstly that *T. brucei* s.l. is probably more widespread than currently implemented surveys based on such molecular tools suggest, both in Western Kenya [15] and elsewhere. Secondly, when designing, and assessing the impact of large scale interventions (eg [25]), the parameters of the collection and testing systems in use must be taken in to account to ensure that appropriate conclusions are drawn and recommendations made. Previous studies may have initially under-estimated the scale of the *T. brucei* s.l. reservoir in different livestock species, and may have under-estimated the impact that mass treatment activities have had in addressing it. Regarding the potential reservoir of zoonotic *T.b. rhodesiense*, cattle and pigs may be a more substantial risk than previously estimated [26] highlighting the need to specifically consider the test parameters for the *T.b. rhodesiense*-specific PCR protocols [14], [27] in future studies. Finally, it is imperative to standardise protocols or establish, as we do here, the relative performance of different protocols across study populations and between testing centres, in order to make meaningful comparisons between different studies. This has largely been acheived for other diseases where standard protocols with known parameters exist [28] but is lacking for a large number of non-reportable infections such as *T. brucei* s.l.

The *T. brucei* s.l. prevalence estimated from PCR results can be adjusted for the test sensitivity and specificity. However, to obtain an equal precision of estimate for the prevalence, a larger number of samples would be required when using the ITS-PCR as compared to the more sensitive *T. brucei* s.l. PCR. For example, at a population prevalence of 10%, 23% more samples would be required when employing ITS-PCR than when employing the specific PCR, to achieve the same absolute precision of 5% (Figure 4). Particularly for large scale studies involving several thousand individual animals, this has implications on the costs and benefits of diagnostic test choice. While our study suggests that the ITS- PCR is less sensitive than the species-specific primers, it is able to simultaneously screen samples for other pathogenic trypanosomes [2]; in multi-organism studies, it may be, overall, a better choice of test.

## Methods

### Ethical Statement

This study used biobank samples of blood from a number of livestock species collected from the ear vein. This non invasive approach requiring minimal restraint of the animals was approved by both the University of Edinburgh ethics review committee and the Kenyan Department of Veterinary Services.

### Study Sites

The samples were collected at two study sites within Busia District, Western Province, Kenya. Site 1, located in Funyula Division, comprised nine adjacent villages. Site 2, located in Butula Division, comprised ten adjacent villages. These two sampling areas were established field sites, originally chosen on the basis of a cattle trypanosomiasis prevalence of at least 6%, as established by a cross-sectional survey in 1997 [29] and were well characterised in terms of livestock-keeping dynamics and veterinary care seeking behaviour [30], [31].

### Sampling

Census sampling targeting the entire livestock population of the two sampling sites was performed in July (Funyula site) and October (Butula site) 2004, by visiting all livestock keeping homesteads in all 19 sampling villages. Whole blood samples from ear-veins were collected from all cattle (n = 1260), pigs (n = 311), goats (n = 764) and sheep (n = 427) at each livestock keeping homestead. A total of 2762 livestock samples from 549 livestock-keeping homesteads were collected. The samples (100 µl) were directly applied to FTA® Cards (Whatman, Maidstone, Kent, UK) and allowed to air dry prior to storage at room temperature, an established method of preservation for sensitive detection of trypanosome infections by PCR [9].

### Laboratory Analysis

Laboratory analysis of all samples was carried out by B.v.W. in the course of her PhD, after one year of laboratory training by K.P..

### Sample Preparation and PCR

All blood samples were analysed by two Polymerase Chain Reactions (PCR) according to the published protocols.For each PCR reaction one 2 mm punch was cut from the samples on the FTA ® Card and prepared according to the manufacturers instructions. Briefly, the discs were washed twice in FTA purification reagent to remove PCR inhibitors from the sample, followed by two washes with 1xTE buffer to remove residual FTA purification reagent. Once dried, the discs were transferred to PCR tubes to seed the reactions.

The first PCR targets the internal transcribed spacers (ITS) located within the ribosomal RNA genes (200 copies/genome) and detects and differentiates between the important pathogenic African trypanosome species affecting livestock, including *Trypanosoma brucei* s.l. [2]. The second PCR employed is specific for *T. brucei* s.l. with a satelite DNA target (10,000 copies/genome) [1]. One positive control (genomic DNA) and one negative control (blank FTA disc) were run with each set of reactions. PCR products were separated by electrophoresis in a 1.5% (w/v) agarose gel containing 0.5 µg/ml ethidium bromide and visualised by ultraviolet light.

### Statistical Analysis

The Hui-Walter paradigm requires two (or more) tests evaluated in two (or more) populations. This model assumes that: (i) the prevalence of the disease is different within each population; (ii) the tests have the same properties across populations; and (iii) the tests are conditionally independent given the disease status. This Bayesian implementation of the Hui-Walter model [32] assumes that for the *i*th sub-population the counts (**O_i_**) of the different combinations of test results, +/+, +/−, −/+ and −/− for the two tests, follow a multinomial distribution:
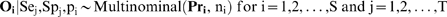
where S is the number of subpopulations, T is the number of tests and **Pr_i_** is a vector of probabilities of observing the individual combinations of test results. Conditioning on the (latent) disease status, these probabilities can be specified using the Se_j_ and Sp_j_ of the tests and the prevalence (p_i_) of the sub-populations. The probabilities of observing each test combination in the *i*th subpopulation are given by:
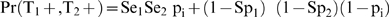


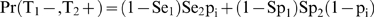


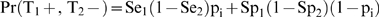


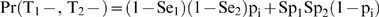



In a Bayesian analysis all parameters are expressed as random variables. Prior distributions for the test properties (sensitivity and specificty) and the prevalence within the sub-populations must be specified. The sensitivity of the two tests and the prevalence in four species were given flat (Uniform(0,1) priors) as there were no published data to inform these estimates. Each test's specificity was given a uniform prior over the range 0.5–1.0. This assumption is still vague but by constraining the specificity above 0.5 we control the label switching issue of Markov chain Monte Carlo (MCMC). This issue is discussed in detail with reference to the estimation of Hui-Walters models by Toft *et al.* [32]. In order to explore the influence of the large catle sub-population the model was re-estimated using only the small ruminant and pig data.

The model was estimated using the JAGS software [33] using the Runjags package [34] of the R statistical system [35] Three MCMC chains were run for this analysis. The first 500,000 iterations were discarded as a burn-in and the following 500,000 iterations were kept and thinned to 50,000 for posterior inference. Convergence of the chains after the initial burn-in was assessed by visual inspection of the time-series plots for the parameter samples as well as Gelman-Rubin diagnostic plots using three sample chains with dispersed starting values [36]. The R package CODA [20] was used for analysis and graphing of the McMC output.

The impact diagnostic test performance can be illustrated by estimation of predictive values, the probability of a tested individual having a given infection status condition on its test result. The positive and predictive values are given by the following formulae:
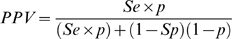
(equation 1)

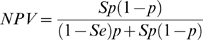
(equation 2)


Where Se is the estimated test sensitivity, Sp is the estimated test specificity, P is the true seroprevalence in the population.

In a classical analysis the single, point estimates of the diagnostic test's sensitivity and specificty are used in these estimators. The Bayesian implementation of the Hui-Walters model produces full, joint posterior estimates of these parameters. We estimated the predictive values of the diagnostic test results over a range of prevalences by numerically integrating equation 1 and equation 2 over the paired estimates of sensitivity and specificity from the model. This approach incorporates the uncertainty and covariance structure of the test performance into the predictive values.

This methodology was extended to estimate the positive and negative predictive values of the two tests in the four study sub-populations by numerically integrating over the joint posterior distribution of prevalence and test sensitivity and specificity for each test/population combination. It is likely that a major application of these tests will be in large scale surveys for the estimation of infection prevalence. Estimates from such surveys will be uncertain due to sampling and imperfections of the diagnostic test. Conventionally these surveys are designed to estimate infection prevalence to a required precision [37]. The required sample size in a survey is a function of the expected prevalence, the diagnostic test performance and the required precision. To estimate the impact of the different diagnostic test performances we calculate approximate sample sizes that would be required for a simple survey over a range of prevalences using the two tests (for a given precision). The analysis uses the following formula for sample size [37] to calculate approximate samples sizes for a 95% confidence with a given absolute error. 
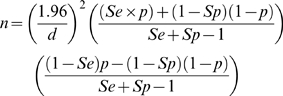



Where Se and Sp are the diagnostic test sensitivity and specificity, p is the expected prevalence and d is the absolute proportional error.
